# Febrile Seizures in an App-Based Children’s Fever Registry: Mixed Methods Study

**DOI:** 10.2196/74933

**Published:** 2026-03-06

**Authors:** David Martin, Ricarda Möhler, Christopher B Germann, Moritz Gwiasda, Silke Schwarz, Ekkehart Jenetzky

**Affiliations:** 1 School of Medicine Faculty of Health Witten/Herdecke University Witten Germany; 2 Department of Pediatrics University of Tübingen Tübingen Germany; 3 Department of Child and Adolescent Psychiatry and Psychotherapy University Medical Center of the Johannes Gutenberg University Mainz Mainz Germany

**Keywords:** children, fever, seizure, ecological momentary assessment, registry, mHealth

## Abstract

**Background:**

Febrile seizures, although typically benign, can cause significant emotional distress for parents. Their diverse etiological risk factors underscore the need for further research. Ecological momentary assessment (EMA) offers a cost-effective and timely method for real-time data collection. The FeverApp, an EMA-based registry for fever management, enables parents to document febrile seizures as they occur.

**Objective:**

This study systematically investigates febrile seizure records from the FeverApp registry to assess their characteristics and explore the clinical implications of the findings. By providing real-world data on seizure management, this research demonstrates the potential of app-based EMA in pediatric care. Additionally, it offers insights for targeted interventions and improved febrile seizure management.

**Methods:**

We used a mixed methods approach. A descriptive qualitative analysis of parental descriptions of 226 seizures belonging to 161 children was conducted. Additionally, a comparative quantitative analysis of group differences was assessed through matched-pair sampling, comparing 114 children. Statistical methods were tailored to the nature of the respective variables, which included prevalence, age, gender, health and febrile history, fever management, temperature, well-being, and parental confidence.

**Results:**

Qualitative analyses provided detailed descriptions of seizure symptoms, seizure duration, and seizure management practices. Additionally, the data revealed a high rate of emergency consultations related to febrile seizures. However, there was underreporting of febrile seizures within the FeverApp, with a reported incidence of only 0.4% among febrile children. In a matched sample controlled for gender and age, significant differences were observed between febrile children with and those without febrile seizures in several parameters, including maximum recorded temperature (*P*<.001), prevalence of chronic diseases (*P*=.004), parental confidence (*P*=.01), and frequency of emergency consultations (*P*<.001).

**Conclusions:**

This study offers valuable insights into the characteristics, temporal dynamics, management strategies, and parental responses to febrile seizures in children. Despite the limitation of potential underreporting in an EMA-based registry, the findings highlight the critical importance of parental education and support in managing febrile seizures. Enhancing these areas has the potential to reduce unnecessary medical consultations and improve the overall care of affected children. Furthermore, integrating improvements in the FeverApp’s education and documentation system regarding febrile seizures could facilitate better management and support future research efforts.

## Introduction

### Theoretical Background

#### Febrile Seizures

Febrile seizures are among the most common pediatric neurological events, affecting 2%-5% of children aged 6 months to 5 years, with a peak at 18 months [1-5]. They occur in the context of febrile illness and are not caused by intracranial infection or central nervous system damage [6,7]. Diagnostic criteria, as defined by the World Health Organization (WHO) *ICD-11* (*International Classification of Diseases, 11th Revision*), are detailed in Multimedia Appendix 1 [8,9]. They are classified as either simple or complex, with the majority being simple (75%-80%) [7,10]. Seizures typically resolve spontaneously, with children recovering within 24 hours, especially in simple cases [4]. They usually occur within the first day of fever, are more frequent in the early evening, and peak in fall and winter [1,7].

The prognosis for children with febrile seizures is generally favorable, with brain damage or death being extremely rare [11,12]. While most children recover without complications, those with complex seizures, a family history of seizures, or younger age have a slightly higher risk of afebrile seizures or later epilepsy [13-15]. Febrile seizures recur in 30%-50% of cases, with recurrence risk increasing with additional risk factors such as lower fever or early onset after fever [7,15,16]. Despite their benign nature, febrile seizures are clinically significant due to their prevalence and potential for recurrence or later epilepsy [4].

Febrile seizures cannot be prevented with antipyretics [17]. Acute seizures can often be stopped with rectal diazepam or buccal/intranasal midazolam [1], while paracetamol and ibuprofen may relieve discomfort but are not recommended solely to reduce fever [18]. Long-term anticonvulsant therapy does not prevent recurrences or later epilepsy [1].

The exact mechanisms underlying febrile seizures remain unclear. Risk factors include a positive family history, certain genetic predispositions, young age (6 months-5 years, peak 12-18 months), developmental delay, neurological abnormalities, prolonged neonatal hospitalization, and maternal smoking [1,4,19,20]. Boys are slightly more affected [6,13,20-22]. Febrile seizures are often associated with infections and may rarely follow vaccinations [4,23-26]. Seizures are typically triggered by elevated body temperature, frequently above 39 °C, though even mild fever can provoke them. It remains unclear whether absolute temperature or the rate of increase is more critical, and seizures can sometimes occur before or after the fever spike [1].

#### Parental Experience and Alleviation

Febrile seizures, though generally benign, are often frightening and distressing for parents, who may fear their child is dying [5,27,28]. Reactions can include psychosomatic symptoms (eg, sleep disruption and dyspepsia) and psychological stress such as anxiety, fear of recurrence, and excessive worry. This can disrupt family life and contribute to “vulnerable child syndrome,” where parents perceive their child as unusually susceptible to problems [5,29]. Despite these fears, children with febrile seizures do not use more medical resources than peers [5]. Studies indicate that many parents lack prior knowledge: in Germany, around 50% had not informed themselves, only 32% recognized a seizure during the episode, and 63% administered antipyretics at lower-than-recommended temperatures [30].

Early recognition, education, and guidance are key to alleviating parental anxiety and improving outcomes. Multimedia educational interventions about the benign nature and management of febrile seizures can further support parents [13]. Health care providers should address misconceptions, fears, and coping strategies to empower parents effectively [5].

This highlights the potential of digital health applications, which can provide real-time guidance, educational support, and data collection to assist families in managing febrile illnesses safely.

#### About the FeverApp

The FeverApp is one of 6 model registries funded by the Federal Ministry of Education and Research (BMBF) and launched in 2019 [31]. It serves as an ecological momentary assessment (EMA) for fever management in families, where parents can document febrile illnesses in real time and receive evidence-based information on fever management [31,32]. Initially, access to the FeverApp was only possible via codes provided by pediatricians. This approach ensured data reliability and accuracy, fostered parental trust, and secured acceptance among treating pediatricians. Since August 2022, the app has been accessible without a code as well.

It is primarily a research tool rather than a certified medical device. Its main purpose is observational, collecting longitudinal data on fever and parental management. At the same time, by monitoring their child’s illness and accessing evidence-based guidance, parents learn about fever, are supported in their care decisions, and may experience reduced anxiety. Its EMA-based design provides clinicians with structured, high-resolution data that can support diagnosis and management, even though the app itself does not make medical decisions.

Technically, the app operates as a client–server system: anonymized data are stored locally in an open-source database (PouchDB) and synchronized with central CouchDB and transferred to MongoDB servers at the University of Witten/Herdecke. The app is available for iOS and Android. It is also possible to extend the functions of the app by implementing additional modules to document specific diseases. In the current app version, it is possible to document febrile seizures separately. Previous studies show that the backend reliably captures real-time data and complements outpatient care [33,34]. A comparison between different fever apps was presented in Joosen et al [35]. The authors reviewed pediatric fever apps for quality and guideline adherence. Out of 878 apps, 3 were fully assessed: Kinsa and FeverApp scored highest in quality, while FeverFriend best adhered to the National Institute for Health and Care Excellence (NICE) guidelines [36]. FeverApp was originally based on the NICE Guidelines until the first author of the present publication and inventor of the FeverApp initiated and coordinated the new German S3 national guideline on fever management in children and youth [37]. The study shows few evidence-based apps exist, including the FeverApp, and highlights their potential for parental support.

### Goal of the Study

Febrile seizures represent a common, most benign yet complex pediatric neurological condition with unknown, probably multifactorial etiology and can be frightening for parents. Understanding these theoretical aspects is critical for developing targeted interventions and providing comprehensive care for affected children and their families.

The quality of data collected is often limited due to reporting and recall bias. Therefore, immediate records are preferred in medical research. EMA is a modern method that enables timely, direct, and cost-effective data collection by allowing repeated sampling of participants’ behavior in real-time environments, thus minimizing recall bias and increasing external validity [38-40].

The primary objective of this study is to investigate the FeverApp as an EMA-based registry for febrile illnesses, with a particular focus on differentiating specific conditions such as febrile seizures. Additionally, the study aims to examine febrile seizure records for their conformity with current research, assess the potential for expanding existing studies, and identify areas for improvement in EMA-based registries.

## Methods

### Data Source

**Figure 1:**
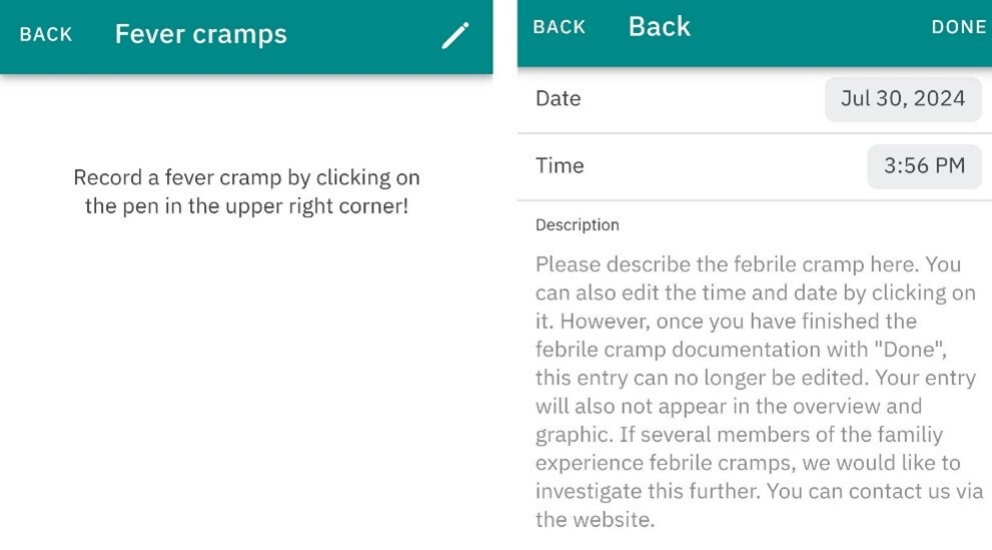
Febrile seizure module within the FeverApp.

**Figure 2:**
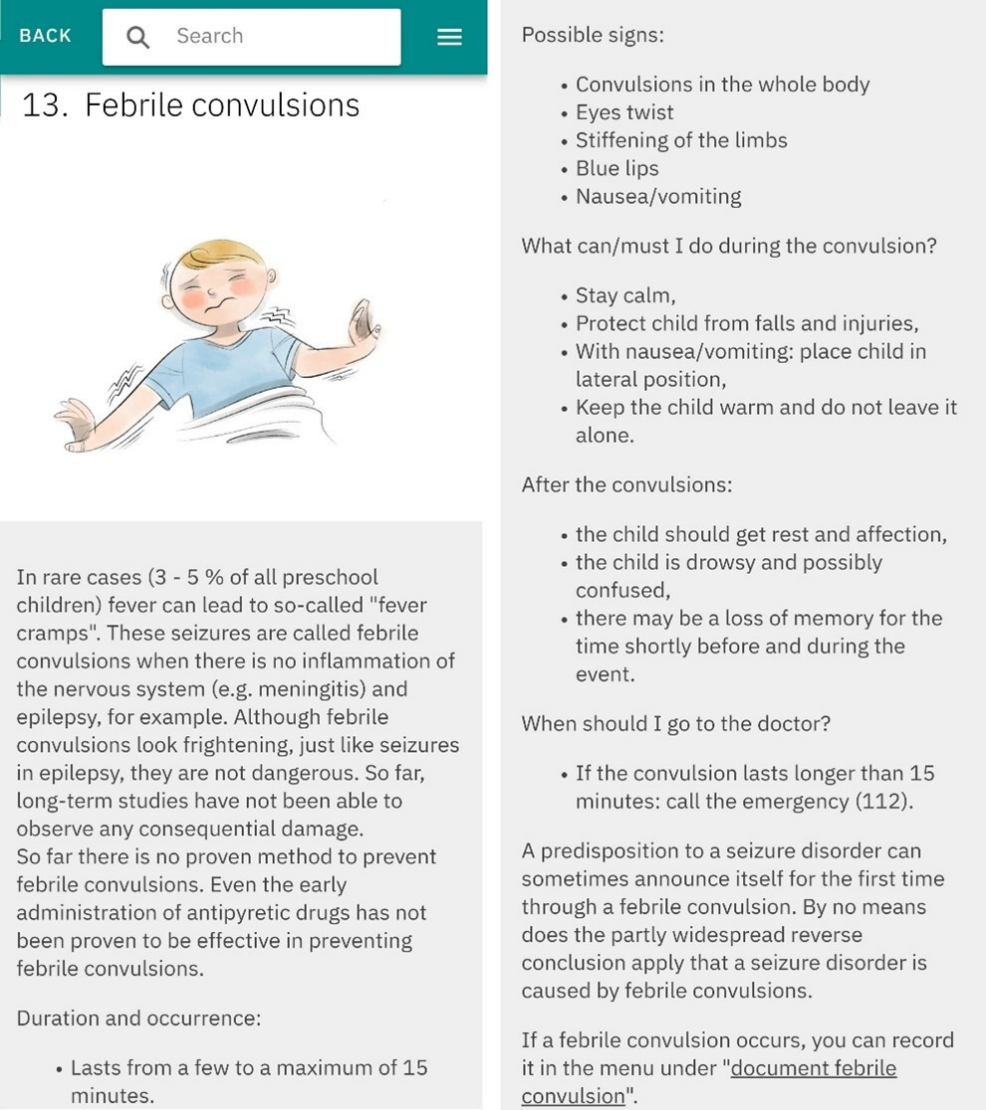
Chapter about febrile seizures in the Info Library of the FeverApp.

The FeverApp serves as an EMA-based registry for fever management, enabling parents to record febrile illnesses and receive advice on managing their child’s fever at home [31]. Since its launch in September 2019, the app has recorded approximately 54,300 fever episodes from around 46,600 children as of November 2024. Data are securely stored on university servers at Witten/Herdecke for scientific research purposes. The app supports various caregiver roles and allows for the creation of multiple profiles and episodes per child. A fever episode concludes either when the parent selects the “child is healthy” option within the app or, during data cleaning, when no further entries are recorded within a 48-hour period. The app collects comprehensive data, including sociodemographic details, medical history, and acute fever-related information such as parental confidence in dealing with their children’s fever, the child’s temperature, symptoms, well-being, and medication usage [32]. Febrile seizures are documented separately, with automatic timestamps and the option for manual adjustments to the date and free-text seizure descriptions (Figure 1). As a continuously updated registry, the completeness of the data varies. Additionally, users have access to educational resources on childhood fever, including a video and a multimedia, guideline-oriented information library with 23 chapters, one of which is dedicated to febrile seizures (Figure 2).

### Recruitment and Sampling

The FeverApp registry constitutes at the beginning a convenience sample of parents who voluntarily use the app to document their child’s fever episodes. Initially, access to the app was provided exclusively through pediatric offices, where physicians distributed access codes to families, ensuring reliability and authenticity of user data. Over time, the app has become publicly available, allowing any interested caregiver to participate voluntarily. Thus, data originates from a naturalistic sample of parents documenting fever episodes in real-life settings.

For this study, no additional recruitment was performed. Instead, a purposive subsample was extracted from the existing FeverApp database, consisting of all cases in which parents documented at least one febrile seizure episode. Entries were included if the seizure documentation appeared reliable, while duplicates or clearly false entries excluded (15 cases). For comparative analysis, a convenience sample of all existing fever episodes were used. In both analyses, children aged 18 years or older, parents who deleted their account, or parents aged 14 years or younger were filtered out. No further exclusion criteria, such as family history of seizures or previous seizures of the child, were applied, as such information was not systematically available.

### Data Analysis

The study followed 2 separate analyses: a descriptive qualitative analysis of parental descriptions regarding febrile seizures and a comparative quantitative analysis between children with and those without a febrile seizure.

Two datasets were used for analysis. Continuous EMA data for each entry were stored in a single large dataset, while a separate dataset was created specifically for time-lagged febrile seizure documentation. Data were subsequently cleaned, processed, and analyzed using IBM SPSS (version 28; IBM Corp).

Initial qualitative analyses focused on entries within the “seizures” dataset. A quantitative analysis of sample characteristics was performed, examining variables such as gender, age, number of febrile seizures recorded, prevalence of chronic disease, febrile disease in the past 12 months, tendency for high fever, typical fever duration, behavior during fever rise, antipyretic use in the past 12 months, time of febrile seizure, and difference between seizure date and seizure record. Some variables (age, febrile disease in the past 12 months, tendency for high fever, typical fever duration, time of the febrile seizure, and difference between seizure date and seizure record) were categorized or dichotomized. Detailed categories are provided in Table 1. Parents’ free-text descriptions of seizure events were manually analyzed following an inductive thematic approach as described by Braun and Clarke [41], aiming to identify recurring patterns, words, and themes that reflected parents’ observations and experiences during febrile seizures to develop categories. To support the interpretation of the qualitative themes, limited frequency indications are provided to show how often specific symptoms or parental descriptions occur. In addition, a quantitative overview of these categories was generated, indicating, for example, the frequency of specific observations such as blue lips or the involvement of emergency services. These descriptive patterns were then interpreted to highlight clinically and behaviorally relevant features of febrile seizures as perceived by parents, such as common symptom clusters, typical parental responses, and indicators of perceived severity, providing insights into both the objective and subjective aspects of seizure events as recorded in a naturalistic setting.

Quantitative analyses within the “seizure” dataset examined variables such as age, gender, number of seizures, health and febrile history (eg, previous fevers, tendency for high fever, typical fever duration, and chronic diseases), fever management strategies (eg, cooling/warming methods and use of antipyretics), timing of seizures, and the time interval between seizure occurrence and documentation.

In the subsequent step, both datasets were merged to incorporate missing variables from the seizure dataset and enable comparisons between febrile children with and those without febrile seizures. As febrile seizures can be recorded some time after they occur, data from fever episodes are not always directly connected. Therefore, seizures in which the timestamp of the entry and the reported seizure differed by more than 48 hours were excluded from the comparison. Since a single child can have multiple fever episodes, comparing children is challenging due to dependency in some entries (multiple episodes for one child) and independence in others (single episode per child). To address this, only the first fever episode for each child was selected for analysis, and in cases of children with seizures, only their first recorded seizure was considered. To control for significant differences in age and gender distribution between the 2 groups, children were randomly matched based on these variables. The analyses examined children with and those without a febrile seizure and compared variables such as age, sex, febrile history (eg, previous fevers, tendency for high fever, and typical fever duration), fever management strategies (eg, cooling/warming methods and use of antipyretics), temperature, parental confidence in dealing with their children’s fever (5-point Likert scale from “thumbs down” to “thumbs up”), medical consultations, and children’s well-being (5-point Likert scale from “sad face” to “happy face”). Some Variables (fever in the past 12 months, tendency for high fever, typical fever duration, and management during fever rise) were categorized or dichotomized. Statistical tests were applied as follows: the *t* test for normally distributed metric data, the Mann-Whitney *U* test for nonnormally distributed metric data (including Likert scale variables, per scale [42]), and chi-square tests for ordinal and nominal data. For 2×2 contingency tables or when cell frequencies were below 5, Fisher exact test was reported. Effect sizes were calculated for all applicable tests to quantify the magnitude of observed differences.

Reporting of this study followed the STROBE (Strengthening the Reporting of Observational Studies in Epidemiology; for quantitative analyses) and SRQR (Standards for Reporting Qualitative Research; for qualitative analyses) guidelines [43,44]. Completed checklists are available in Multimedia Appendices 2 and 3 [43]. While the study includes qualitative analysis of parental free-text entries regarding febrile seizures, it does not follow traditional qualitative research design (eg, interviews or ethnography). Therefore, some items from the SRQR checklist are not applicable. The analysis was limited to the content of entries collected in the naturalistic, real-world setting of the FeverApp.

**Table 1:** Descriptive analyses of seizure dataset.

Characteristic		Value, n (%)
**Gender (n=161)**		
	Male	81 (50.3)
	Female	80 (49.7)
**Age (n=161)**		
	0–12 months	24 (14.9)
	13–24 months	51 (31.7)
	2-5 years	72 (44.7)
	>5 years	14 (8.7)
**Number of febrile seizures recorded (n=161)**		
	1	128 (79.5)
	2	20 (12.4)
	3	4 (2.5)
	4	4 (2.5)
	5	1 (0.6)
	6	3 (1.9)
	7	1 (0.6)
Chronic disease present (n=161)		17 (10.9)
**Fever in the past 12 months (n=145)**		
	0	27 (18.6)
	1-5	96 (66.2)
	>5	22 (15.2)
**Tendency for high fever (n=142)**		
	“Never” to “few times”	102 (71.8)
	“Most” to “always”	40 (28.1)
**Typical fever duration (n=44)**		
	≤3 days	43 (97.7)
	>3 days	1 (2.3)
**Behavior during fever rise (n=95)**		
	Cooling	30 (31.6)
	Warming	20 (21.1)
	Neither	29 (30.5)
	Don’t know	16 (16.8)
Antipyretics in the past 12 months (n=140)		114 (81.4)
**Time of the febrile seizure (n=226)**		
	5 AM-10 AM	43 (19.0)
	11 AM-4 PM	81 (35.8)
	5 PM-11 PM	83 (36.7)
	12 AM-4 AM	19 (8.4)
**Interval between seizure date and record (n=226)**		
	<24 hours	127 (56.2)
	<1 week	34 (15.0)
	<1 month	14 (6.2)
	>1 month	28 (12.4)
	>6 months	23 (10.2)

### Ethical Considerations

Ethical approval for the FeverApp registry has been granted by the ethics committee of the University of Witten/Herdecke (proposal number: 139/2018).

The FeverApp Registry is registered in the German Clinical Trials Register (DRKS-ID: DRKS00016591). The registry follows the Declaration of Helsinki. Informed consent was obtained from all participants involved in the study. After installing the app, users must confirm a data privacy statement where they accept that their data are being recorded and used for scientific reasons. The FeverApp ensures privacy and confidentiality through data anonymization. Each family is assigned a randomly generated 8-character code, and all users and children are identified only by these codes. Data can be deleted at any time either via the app or by contacting the study team by email to request data removal. No compensation was provided to participants, as the study was based on data collected through the FeverApp and did not involve direct participation or intervention.

## Results

### Dataset of Febrile Seizures

**Figure 3:**
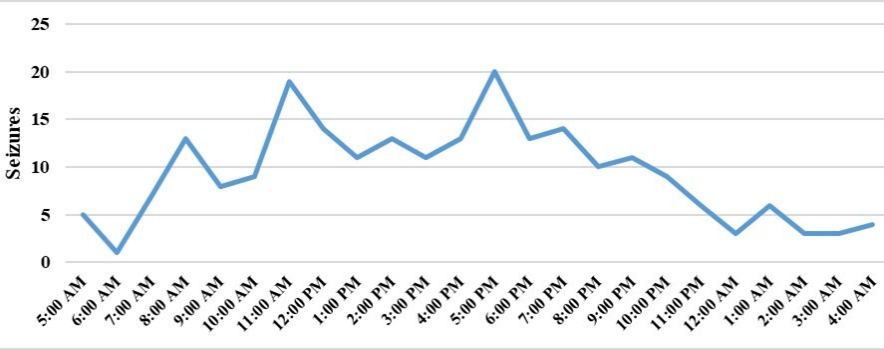
Seizure frequencies at different times.

Over a data collection period of 5 years, there were 226 registrations of febrile seizures. These belonged to 161 children, of whom 128 (79%) had only 1 febrile seizure and 33 (21%) had 2 or more febrile seizures. From 226 registered febrile seizures, 125 (60%) included detailed descriptions. Children’s gender was evenly distributed. A chronic disease was present in 10% (17/156) of the children. Regarding fever history, around 81% (118/145) of children had experienced a febrile illness in the past 12 months. Most children (102/142, 72%) rarely or never have high fever, and nearly all (43/44, 98%) have a typical fever duration of up to 3 days. Parents’ responses varied regarding their actions during a fever rise, with 21% (20/95 children) warming, 31% (30/95 children) cooling, and 31% (29/95 children) doing neither. In 81% (114/140) of children, antipyretics were administered within the past 12 months. Approximately 15% (24/161) of the children were aged 12 months or younger, while about one-third (51/161, 32%) were aged between 12 and 24 months. Nearly half (72/161, 45%) of the children were aged between 2 and 5 years, and approximately 8% (14/161) were aged 5 years or older. Most records of febrile seizures were between 8 AM and 8 PM (168/226, 74%), with peaks at 11 AM and 5 PM (Figure 3). In most cases (127/226, 56% records), the time between the seizure and its recording was less than 24 hours, although in around 10% (23/226 records) of cases, parents recorded the seizure more than 6 months after it happened. Additional results are provided in Table 1.

### Descriptive Qualitative Analysis of Parental Descriptions of Febrile Seizures

A total of 70 children had 1 detailed description, and 17 children had 2-6 descriptions of their seizures. They are reported separately because of their possible different seizure profile.

#### Children With 1 Seizure Report

High fever was reported in 13 of 70 cases, with temperatures ranging from 39.1 °C to 40.2 °C. Some described it as “sudden fever,” “fast rising,” or “from 0 to 100.” Two of 70 parents reported lower temperatures from 38.0 °C to 38.8 °C. The duration of the seizures ranged from 30 seconds to 10 minutes (40/70), with the majority lasting less than 3 minutes (26/70). One parent reported that the seizure lasted for about half an hour.

Out of 70 descriptions, some parents described their child as whiny (3 cases) or restless, lethargic, clingy, and tired (2 cases each) before the seizure. During the seizure, parents frequently reported “rolling eyes,” that is, upward gaze deviation (29 cases), cramping (25 cases), twitching (24 cases), tension or stiffness (15 cases), blue lips (15 cases), unresponsiveness (15 cases), and salivating (9 cases). Additional symptoms included a staring gaze (6 cases) and blue (5 cases) or pale (3 cases) skin color. Some parents reported rhythmic movements (5 cases) and others a limp child (4 cases). Children whimpered or cried (4 cases), screamed (4 cases), vomited (4 cases), and clenched or ground their teeth (3 cases) during the seizure. Breathing was described as gasping for air (6 cases), wheezing (3 cases), or flat breathing (2 cases). Tachycardia was infrequently reported (2 cases). In 3 cases, the seizures were described as uncomplicated and quickly resolved. One mother reported that she was “scared” and “thought he was dying.”

After the seizure, 5 out of 70 cases reported that the child was not responsive for a period or that they fell asleep immediately. In 3 cases, the child vomited afterward. Five of 70 parents reported that their child seemed very limp afterward.

In 19 out of 70 cases, an ambulance or emergency doctor was called. One parent waited and then went to the doctor the next morning. In 13 out of 70 cases, the child was taken to the hospital for overnight monitoring or a few days of observation. Diagnoses reported included kidney inflammation, tonsillitis or throat infection, ear infection, and 3-day fever.

Various measures were taken before, during, or after the seizures. In 2 out of 70 cases, antipyretics were given directly before the seizures, and in 5 of 70 cases, they were given afterward. During the seizures, diazepam was administered once, buccolam once, an “antispasmodic medication” once. Additionally, antipyretics (4/70 cases) were administered during the seizure. Other measures included calf wraps, cooling of the forehead and legs (2 cases), removing clothes (1 case), and placing the child in a stable side position (3 cases).

Overall, these reports illustrate the range and frequency of symptoms, postictal behaviors, and parental responses during single febrile seizure events, highlighting commonly observed patterns such as high fever, eye rolling, cramping, cyanosis, and emergency interventions.

#### Children With More Than 1 Seizure Report

The temperature ranged from 38 °C to 40.1 °C. Many cases (10/56) had a higher fever than 39 °C. Two reports described low or no fever. A fast fever rise was noted in 2 cases. The duration of the seizure ranged from 50 seconds to 30 minutes. Most seizures (20/56 cases) lasted up to 2 minutes. In 9 cases, a duration of 5 to 10 minutes was reported, and in 3 cases, a duration of 20 to 30 minutes was reported.

In some cases (6/56), parents described that their child was “not really responsive” or “not quite himself” before the seizure even started. Parents described their child as very sleepy (4 cases) and that the child had fever throughout the day (3 cases). Twitching, lots of crying, being shaky on the legs, and screaming (2 cases each), as well as restlessness and stretching (1 case each), were also reported before the seizure. One parent noted that there were no signs of fever or an infection before the seizure.

Among the 56 documented seizures, common clinical manifestations included tonic-clonic activity (cramping, 27 cases), oculogyric crisis (fixed gaze, 18 cases), perioral cyanosis (blue lips, 14 cases), postictal state or loss of consciousness (13 cases), and myoclonus or clonus (twitching, 11 cases). Additionally, a tonic gaze or ictal fear was noted in 11 cases. Less frequent findings included ictal vocalizations (screaming, 9 cases), acrocyanosis or pallor (blue or pale skin, 6 cases), and emesis (vomiting, 4 cases). Some children exhibited opisthotonos (abnormal posturing, 3 cases), rigidity or hypertonia (increased muscle tone, 2 cases), hypotonia or flaccidity (decreased muscle tone, 2 cases), or automatisms (stereotyped rhythmic movements, 1 case). Respiratory compromise was observed in several cases: apnea (cessation of breathing) or dyspnea (difficulty breathing) was reported in 3 instances, and expiratory stridor (wheezing) in another 3. One case involved significant oxygen desaturation requiring supplemental oxygen therapy. Normal respiration was explicitly documented in only 1 case. Regarding seizure characteristics, 2 episodes were described as uncomplicated (simple febrile seizures), while 3 were noted to resemble prior seizures (recurring febrile seizures). One parent expressed acute psychological distress, describing the event as “the horror.”

In 10 out of 56 cases parents reported that their child remained not responsive and required 20 to 30 minutes to be “back to himself/herself.” In 1 case it took over 45 minutes and sleep for the child to become responsive again. Two cases reported that the child was not able to talk for a while. In many cases (n=17), children were very sleepy or lethargic afterward. Parents reported that their child was vomiting (2 cases), salivating (1 case), screaming (1 case), and twitching (1 case) afterward. One noted that she had “the feeling the seizure continued.”

In 16 out of 56 cases, an ambulance was called, or they drove to the emergency room, and in 15 cases, they stayed at the hospital for observation for up to 2 days. One parent was going to a neurologist the next day. Influenza A (2 cases), a side effect of the measles vaccination (1 case), and a mild blood infection (1 case) were diagnosed in the hospital.

Paracetamol was given before the seizure in 1 case, and in 2 cases, paracetamol and ibuprofen were administered. In many cases (16/56 cases), diazepam was given during the seizure. In 2 cases, a double dose of diazepam or diazepam and additionally buccolam was administered. In 1 case it was a combination of diazepam and an antipyretic. Some children (3 cases) received paracetamol or ibuprofen afterward as well. In contrast to children with only 1 seizure or their first seizure, children with more seizures received diazepam as an anticonvulsive medication more frequently.

Overall, these reports illustrate the range and frequency of symptoms, postictal behaviors, and parental responses during multiple febrile seizure events, highlighting recurring patterns such as tonic-clonic activity, blue lips, postictal unresponsiveness, and the frequent involvement of emergency services or administration of diazepam.

### Comparative Quantitative Analysis of Febrile Children With or Without a Febrile Seizure

#### Overview

Only 0.4% of all children in the FeverApp had a febrile seizure documented. Although most children with a fever were male (14,298/27,342, 52.3%), febrile seizures were evenly distributed (57 female, 57 male; total 114) around gender. The median age of children with a febrile seizure was 30 months (IQR 13-42) and without a febrile seizure was 21 months (IQR 7-36). The age distribution between the groups differed significantly in the 2-tailed *t* test (t_27237_=–2.41, *P*=.02). After matching, each group consisted of 114 children with an identical distribution of age (mean 31.68, SD 24 months) and gender (57 male and 57 female children in each group).

#### Temperature

Children’s mean temperature without a febrile seizure was 38.6 °C and ranged within an episode from 38.0 °C to 39.0 °C. The mean temperature with a febrile seizure was 38.7 °C and ranged from 37.9 °C to 39.5 °C. The difference was significant in the Mann-Whitney *U* test between the highest reported temperature during an episode, with a moderate effect (*U*=2915; *z*=–4.25; *P*<.001; *r*=–0.25).

#### Parental Confidence

**Figure 4:**
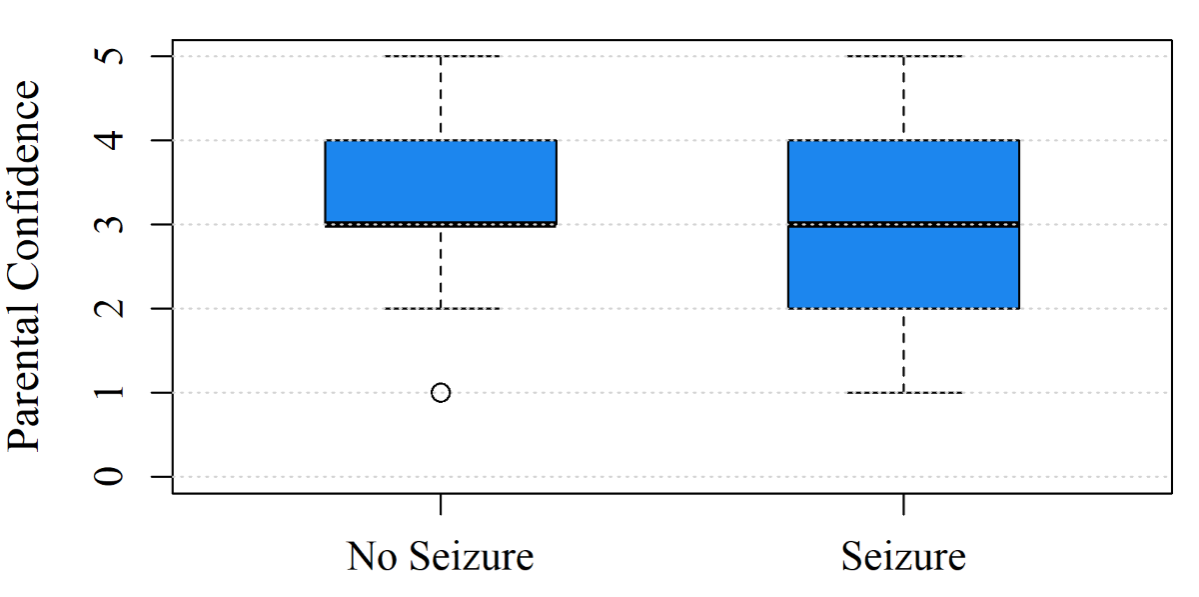
Boxplot comparing the minimum parental confidence levels between the group of children with and without a seizure.

A Mann-Whitney *U* test was conducted to compare the minimum and maximum reported parental confidence during an episode between parents of children with and those without a febrile seizure. Although the medians were identical in both groups (minimum median 3, IQR 3-4; maximum median 4, IQR 3-5), the average lowest reported confidence differed: parents of children with a febrile seizure reported an average minimum confidence level of 2.9, while parents of children without a febrile seizure reported an average of 3.5. The test revealed a significant difference in the distribution of minimum parental confidence with a small effect size (*U*=3657; *z*=–2.45; *P*=.01; *r*=–0.18). As illustrated in the boxplot in Figure 4, this difference may be explained by the greater spread toward lower values among parents of children with febrile seizures compared with those without.

#### Children’s Well-Being

A Mann-Whitney *U* test was conducted to compare the minimum and maximum reported children’s well-being during an episode between the group of parents with children with and those without a febrile seizure. The medians for both groups ranged between 2 and 3 (low to moderate well-being) within an episode, with no significant differences. Further information is provided in Table 2.

**Table 2:** Results from the comparison of children with and those without a seizure.

Variable		No seizure	Seizure	*P* value	*t* test (df)	Mann-Whitney *U* value	*z* value	Effect size	
								Cohen *d*	Pearson *r*
**Temperature (n=196)**									
	Mean in episode, mean	38.6 °C	38.7 °C	.28	–1.09 (194)	—^a^	—	0.77	—
	Minimum in episode, mean	38.0 °C	37.9 °C	.28	1.09 (168)	—	—	0.99	—
	Maximum in episode, mean	39.0 °C	39.5 °C	<.001	—	2915	–4.25ᶠ	—	0.25
**Parental confidence (n=192)**									
	Minimum in episode, median (IQR)	3 (IQR 3-4)	3 (IQR 2-4)	.01	—	3657	–2.45ᶠ	—	–0.18
	Maximum in episode, median (IQR)	4 (IQR 3-5)	4 (IQR 3-5)	.93	—	4527	–0.09ᶠ	—	–0.01
**Children’s well-being (n=199)**									
	Minimum in episode, median (IQR)	2 (IQR 1-2)	2 (IQR 1-2)	.26	—	4501	–1.13ᶠ	—	–0.08
	Maximum in episode, median (IQR)	3 (IQR 2-4)	3 (IQR 2-4)	.09	—	5584	–1.66ᶠ	—	–0.12

^a^Not applicable.

#### Contact With a Doctor

In 59% (64/196) of cases, parents had no contact with a doctor when their child did not have a febrile seizure, whereas in the group of children with a febrile seizure, only 34% (30/196) had no contact with a doctor. In this group, 35% (31/196) had contact with an emergency service. Parents who did not have a child with febrile seizure only contacted an emergency service in around 5% (5/196) of cases. The difference was significant in the Fisher exact test, with a moderate effect (*P*<.001; Cramer V=0.401).

#### Vaccination in the Past 2 Weeks

About 14% (14/179) of children with no febrile seizure had a vaccination in the past 2 weeks. There was a lower percentage of recent vaccination (8/179, 11%) in the group of children with a febrile seizure, although this difference was not significant in Fisher exact test between the 2 groups.

#### Chronic Diseases

In the group of children with a seizure, 14% (16/203) had a chronic disease, whereas only 3 % (3/203 cases) in the group with no seizure had a chronic disease. Fisher exact test indicated that the difference was statistically significant at the conventional alpha level, with a weak effect (*P*=.004; Cramer V=0.196). Most children had asthma (5 cases), epilepsy (2 cases), or neurodermatitis (2 cases).

#### Fever in the Past 12 Months

Around one-third of children in the group without a seizure (31/101, 31%) and around one-fourth in the group with a seizure (24/101, 24%) had no fever in the past 12 months. Around two-thirds of children in both groups (61/101, 60% vs 62/101, 63%) had fever 1-5 times in the past 12 months. There was no significant difference in the chi-square test.

#### Tendency for High Fever

Almost all children in both groups (83/194, 85% vs 75/194, 78%) had a tendency for high fever “never” to “few times.” There was no significant difference in the chi-square test.

#### Typical Fever Duration

Almost all children in both groups (24/56, 92% vs 29/56, 97%) had a normal fever duration of up to 3 days. There was no significant difference in the Fisher exact test.

#### Antipyretics in the Past 12 Months

Most children in both groups got antipyretics in the past 12 months (79/188, 82% vs 73/188, 79%). There was no significant difference in the chi-square test.

#### Cooling/Warming During Fever Rise

More parents in the group of children with a febrile seizure would cool their child during fever rise (22/106, 42%) than in the group of children with no febrile seizure (15/106, 28%). Around one-quarter of parents in both groups would warm their child during fever rise (15/106, 28% each). Around 30% (16/106) of parents in the group of children with a febrile seizure would neither cool nor warm their child, whereas 43% (23/106) of parents in the group of children without a febrile seizure would do neither. Even though there was a relevant difference in percent between the groups, these differences were not significant in the chi-square test. Table 3 shows further results from the comparison of children with and those without a seizure.

**Table 3:** Results from the comparison of children with and those without a seizure.

Variable		No seizure, n (%)	Seizure, n (%)	*P* value	Chi-square (*df*)	Effect size^a^
**Doctors contact (n=196)**				<.001^b^	—^c^	0.40
	No contact	64 (59)	30 (34)			
	Yes, with their doctor	36 (33)	25 (28)			
	Yes, with a substitute	3 (3)	2 (2)			
	Yes, with the emergency service	5 (5)	31 (35)			
**Vaccination in the past 2 weeks (n=179)**				.57^b^	—	0.04
	Yes	14 (13.5)	8 (10.7)			
	No	90 (86.5)	67 (89.3)			
**Chronic diseases (n=203)**				.004^b^	—	0.2
	Yes	3 (2.8)	16 (13.8)			
	No	103 (97.2)	100 (86.2)			
**Fever in the past 12 months (n=101)**				.45^d^	1.61 (2)	0.90
	0	31 (30.7)	24 (24.2)			
	1-5	61 (60.4)	62 (62.6)			
	Over 5	9 (8.9)	13 (13.1)			
**Tendency for high fever (n=194)**				.24^d^	1.39 (1)	0.08
	“Most” to “always”	15 (15.3)	21 (21.9)			
	“Never” to “few times”	83 (84.7)	75 (78.1)			
**Typical fever duration (n=56)**				.59^d^	—	0.1
	Up to 3 days	24 (92.3)	29 (96.7)			
	More than 3 days	2 (7.7)	1 (3.3)			
**Antipyretics in the past 12 months (n=188)**				.60^d^	0.26 (1)	0.04
	Yes	79 (82.3)	73 (79.3)			
	No	17 (17.7)	19 (20.7)			
**Management during fever rise (n=106)**				.28^d^	2.58 (2)	0.16
	Warming	15 (28.3)	15 (28.3)			
	Cooling	15 (28.3)	22 (41.5)			
	Neither	23 (43.4)	16 (30.2)			

^a^Cramer V.

^b^Fisher exact test.

^c^Not applicable.

^d^Chi-square test.

## Discussion

### Overview

The primary aim of this study was to assess the effectiveness of the FeverApp as an EMA registry for febrile illnesses, specifically in relation to febrile seizures. Additionally, the study sought to expand existing research and identify improvements for associated EMA registries. Our results provide valuable insights into the characteristics and management of febrile illnesses in children.

### Descriptive Qualitative Analysis of Parental Descriptions of Febrile Seizures

The detailed descriptions of febrile seizures provided by parents offer critical insights into the nature and management of these events. Of 226 reported febrile seizures, a significant portion (60%) included comprehensive reports, revealing common patterns in symptoms and parental actions during these distressing episodes. These included temperature, duration, description of the seizure itself, measures that were taken, and contact with a doctor. Some parents even recorded the seizure years after it occurred, which highlights the importance in their perception. The febrile seizure module itself is not in the normal backend of the FeverApp but in a different menu, so users must invest extra cognitive effort to record febrile seizures. This finding likewise emphasizes the importance of precise documentation and longitudinal analyses to better understand the lasting effect of febrile seizures.

Among children with a single seizure (13 out of 70 cases) and those with multiple seizures (10 out of 17 cases), many parents reported a high fever of 39.0 °C or higher immediately preceding the seizure. This observation aligns with current research indicating that 75% of seizures in children occur at body temperatures exceeding 39 °C [1]. Descriptions of the fever as “fast rising” or “from 0 to 100” in children with a single seizure (2 cases) and in those with multiple seizures (2 cases) suggest that rapid increases in body temperature may act as a trigger as well. This observation is consistent with existing literature on febrile seizure [1].

The duration of seizures varied between both groups, with most episodes lasting less than 3 minutes. This finding is congruent with related research, where seizures were reported to typically last 1-1.5 minutes. In both groups, however, seizures extended up to 10 minutes, and in 3 cases even up to 20-30 minutes. This may be due to a temporal overestimation on the part of the parents, or as an indicator that the episode constitutes a more complex seizure. Due to the highly emotional nature of seizures, especially during a child’s first episode, parents’ subjective time perception may often overestimate the actual duration [1,45], that is, this deviation may constitute a systematic time perception bias that warrants further investigation. It should be noted that parents who reported a longer duration consistently described the seizure with additional symptoms (ie, “teeth grinding” and “muscle cramping”). These symptoms were not halted by diazepam, which is indicative of a more complex seizure (prevalence estimated to be 20%-25%) [7,10].

Parents frequently noted a period of postictal unresponsiveness, with some children taking up to 20-30 minutes to recover (in some cases, the child fell asleep). This observation aligns with the typical postictal phase seen in febrile seizures, where children may appear disoriented or lethargic following the event [1].

The symptoms reported by parents, such as rolling eyes, blue lips (cyanosis of the lips), unresponsiveness (altered consciousness or unresponsiveness), twitching, and cramping, are characteristic of febrile seizures and are well-documented in the pertinent clinical literature [46]. The variety of symptoms like smacking, teeth clenching, and vomiting, demonstrates that febrile seizures can manifest in a wide range of symptoms.

In terms of emergency response, the descriptions show parental concern in both groups, with an ambulance or emergency doctor being called in 35 cases. The fact that 28 children were admitted to the hospital for observation can be an indicator either for medical evaluation following a febrile seizure or parent’s anxiety [1]. An inpatient admission is considered depending on the age, the complexity of the seizure, the result of the clinical examination and parents’ anxiety [1]. A further factor is that some hospitals encourage high admission rates for financial reasons.

Antipyretics were administered both prophylactically and reactively, reflecting their widespread use in fever management. It should be emphasized that empirical data indicate that lowering fever does not prevent the occurrence of febrile seizures [17]. In the group of children with more than 1 seizure diazepam was frequently administered. Most febrile seizures are short, typically resolve on their own, and thus do not need immediate pharmacological intervention. However, when a seizure lasts longer than 5 minutes, it may require medical treatment [47]. It is also recommended to prescribe diazepam after the first seizure in case of a relapse, but also for the feeling of security of parents [1]. However, children are often very strongly affected by diazepam, and such pharmaceutical drugs are not for parents “emotional safety.” Benzodiazepines have side-effects (especially in neurosensitive children) and should only be prescribed to children as an ultima ratio [48]. Studies have shown that exposure to benzodiazepines can lead to neuronal apoptosis in the developing brain. For instance, research on neonatal macaques demonstrated that short-term exposure to these drugs resulted not only in neuronal apoptosis but also in oligodendroglial apoptosis, impaired synaptogenesis, inhibited neurogenesis, and subsequent long-term neurocognitive deficits [49]. Our understanding of how benzodiazepines affect the neurosensitive developing brain remains very limited. Research into the GABAergic and opioid systems is evolving, and the interplay between neurochemistry and cognitive development (consciousness) represents a cutting-edge area of study. It cannot be prima facie excluded that benzodiazepines may have longitudinal, and potentially epigenetic, effects on neuronal development [50,51]. This possibility underscores the importance of caution in their use and the exploration of safer and less controversial alternatives. There is a growing awareness of the risks associated with benzodiazepine use in children. The US Food and Drug Administration has updated boxed warnings for all benzodiazepine medicines to highlight the risks of abuse, misuse, addiction, physical dependence, and withdrawal reactions consistently across all drugs in this class [52].

### Comparative Quantitative Analysis of Febrile Children With or Without a Febrile Seizure

The quantitative analysis showed that febrile seizures were reported in only 0.4% of cases in the FeverApp, which is likely an underreporting compared to the 2%-5% incidence documented in the literature [2-4]. While app-based registries can capture certain diseases, recording serious events seems to remain a challenge. The relevant module is in a separate menu of the FeverApp, located along with technical settings and information about the app, rather than being integrated into the main backend. Enhancing the accessibility of this module could lead to more accurate records. Additionally, the app should encourage users to distinguish more precisely between simple and complex convulsions and to note subsequent events and preperceptions which could enhance the understanding of febrile seizures. Lastly, including a video about febrile seizures in the information library could both improve the module’s visibility and provide educational support, increasing user confidence in this area.

Several medical studies and resources document that the risk of febrile seizures is higher in males [13,20-22]. However, the FeverApp shows a slight predominance of female children with febrile seizures, which may also indicate the underreporting within the app. The majority of the children in the study had only 1 recorded seizure, which speaks for a simple nature of the seizure and aligns with reports in the literature [5].

For a better comparison of the 2 groups (viz, children with and those without a febrile seizure), they were matched regarding age and gender. One of the key findings is the significant difference in the highest reported temperatures between children with and those without febrile seizures. This suggests that higher temperatures might trigger a febrile seizure, which is in alignment with current research [1]. On the other hand, children who experienced febrile seizures had a wider range of temperatures, indicating that even a relatively low fever can potentially trigger a seizure. This supports the idea that individual susceptibility plays a role in the occurrence of febrile seizures and that seizure thresholds can vary among children [1]. It has been stated that recurrent febrile seizures and a family history of febrile seizures have a lower threshold in temperature as well [4].

In managing fever, a notable difference was observed in how parents responded to the onset of fever. Parents of children with a history of febrile seizures were more likely to report cooling their child during a fever rise. In contrast, parents of children without such a history were more inclined to take no action or to warm their child during a fever rise. This disparity highlights the heightened concern among parents regarding febrile seizures and their proactive efforts to manage them through cooling. It also emphasizes the importance of providing proper education on febrile seizures and their management strategies. It is to date unclear whether cooling decreases or increases the probability of developing a febrile seizure. Tumor necrosis factor ⍺ and other cytokines may lower the seizure threshold. Given that their increase in fever can, to a certain extent, be neutralized by warming the child, it has been proposed that warming in the phase of a rising fever may prevent febrile seizures [53,54].

Children with a febrile seizure were more likely to have a chronic disease (notably asthma and neurodermatitis) which is found in other studies as well. A relevant study in Taiwan found a risk of subsequent asthma in children with febrile seizures [55], and a study in Iran found that febrile seizures are associated with the number of comorbid allergic diseases in children [56]. A Turkish study found that children have a higher risk for febrile seizures when they have a chronic illness that requires continuous medication [57].

Despite the differences in temperature, the reported well-being of the children did not significantly differ between the groups. This suggests that while febrile seizures are distressing, they do not necessarily correlate with a perceived decrease in the overall well-being of the child, as assessed by the parents. This finding is important as it may help reassure parents that febrile seizures, while emotionally alarming, are generally not indicative of a more serious underlying condition affecting the child’s health.

Interestingly, no significant differences were found between the groups regarding vaccination history in the past 2 weeks, despite the potential for an increased risk of febrile seizures following certain childhood vaccines, such as the diphtheria, tetanus, and pertussis vaccine, as well as the measles-mumps-rubella vaccine [4]. Similarly, there were no notable differences between the groups in terms of fever frequency, duration, or antipyretic use over the past 12 months.

The study also highlighted significant differences in health care use between the 2 groups. Parents of children who experienced a febrile seizure were more likely to seek medical attention, particularly from emergency services. This is consistent with the high level of concern febrile seizures generate, often prompting immediate medical consultation. They usually occur in the evening and thus lead to emergency consultations. In contrast, parents of children without febrile seizures were less likely to contact a doctor, reflecting greater confidence in managing routine fevers at home. This is also shown in the significant difference in confidence levels between the 2 groups. Parents of children who had experienced a febrile seizure reported lower confidence levels. This is also reported in the descriptions, where one parent noted that “it was the horror” and one parent said she was “scared” and “thought he was dying.” The lower confidence and higher emergency consultations among these parents highlight the need for better education and support to help them manage future febrile episodes more effectively.

### Strengths, Limitations, and Future Research

This study has limitations. First, all data were self-reported by parents through manual entry rather than automated measurement or clinical verification, which may introduce recall bias, subjectivity, or incomplete reporting, particularly regarding seizure duration and symptoms. While some physiological parameters (eg, temperature or heart rate) could potentially be captured with automated devices, febrile seizures themselves are complex, short-lived events that are initially observed by parents. Seizures are more standardized in clinical settings, but often occur at home and are initially described by caregivers, which limits full automation of data collection in real-world environments. Second, although we used a purposive sample, the relatively small number of detailed seizure descriptions (73 out of 121 cases) and general underreporting of febrile seizures among febrile children (0.4%), limits the generalizability of the findings and formal statistical inference for the broader population. Finally, early pandemic restrictions and later increases during the Omicron wave influenced app use [32,58], but these effects likely evened out across the full dataset collected through 2023.

Despite its limitations, one of the primary strengths of this study lies in the comprehensive data collected from a large cohort of children using the FeverApp, providing valuable insights into febrile seizures and their management in a real-world setting. The matching of the sample led to a more valid comparison of the 2 groups, with age and gender differences being controlled as well as accounting for underreporting in the FeverApp. The detailed parental descriptions of febrile seizures offer a unique perspective on the symptoms, duration, and immediate responses, which are often difficult to capture in clinical settings. Additionally, the study highlights the variability in fever patterns and seizure characteristics, contributing to a more nuanced understanding of febrile seizures.

Future research could aim to further enhance the data quality of the FeverApp and clinical relevance through partial integration of automated measurements from validated medical devices (eg, temperature or heart rate sensors). Such integration could help to more precisely determine temporal relationships between fever onset, physiological changes, and the occurrence of seizures. In addition, the findings from this study could serve as a basis for developing a structured febrile seizure module with standardized questions reflecting the most frequently reported and relevant anamnestic parameters.

### Conclusions

This study offers valuable insights into the characteristics, management, and parental responses to febrile seizures in children, as documented in the FeverApp registry. Its strengths include detailed parental descriptions and real-world data on fever management practices, contributing to a deeper understanding of febrile seizures. However, the findings also highlight the need for further research into the psychological impact on parents. Despite limitations such as reliance on self-reported data and the lack of clinical verification, the study underscores the critical importance of parental education and support in managing febrile seizures. Enhancing these areas could reduce unnecessary medical consultations and improve the care of febrile children. Future research should investigate the effectiveness of parental education in managing febrile seizures and evaluate whether targeted interventions can reduce reliance on emergency medical services. Additionally, improving the reporting mechanism within the FeverApp to better capture febrile seizure data is warranted, as this could address underreporting and support further research. Although video documentation was omitted due to data safety concerns, the registry-based data provides an excellent foundation for future trials on this important topic.

## Appendix

Multimedia Appendix 1 Febrile seizures: World Health Organization (WHO) International Classification of Diseases, 11th Revision (ICD-11) classification and clinical characteristics.

Multimedia Appendix 2 STROBE checklist for quantitative analysis.

Multimedia Appendix 3 SRQR checklist regarding qualitative analysis.

## Abbreviations

BMBF: Federal Ministry of Education and Research
EMA: ecological momentary assessment
ICD-11: International Classification of Diseases, 11th Revision
NICE: National Institute for Health and Care Excellence
SRQR: Standards for Reporting Qualitative Research
STROBE: Strengthening the Reporting of Observational Studies in Epidemiology
WHO: World Health Organization

## Data Availability

The datasets analyzed during this study are available from the corresponding author on reasonable request.
