# Supplementary material for: Febrile Seizures in an App-Based Children’s Fever Registry: Mixed Methods Study
Source: JMIR Pediatr Parent. 2026 Mar 6;9:e74933. doi: 10.2196/74933 (PMC13005064; doi:10.2196/74933)
Supplement: Multimedia Appendix 1 [file pediatrics_v9i1e74933_app1.docx]

**Febrile Seizures: WHO ICD-11 Classification and Clinical Characteristics**

Febrile seizures, classified under ICD code **8A63.0**,^[[1]](#footnote-1)^ are seizures that occur in conjunction with a rise in body temperature, typically associated with febrile illnesses. They are distinct in that they arise without underlying intracranial infections, metabolic disturbances, or a prior history of afebrile seizures. These seizures predominantly affect children between the ages of **6 months and 5 years**, making them one of the most common neurologic disorders in this age group.

The International Classification of Diseases (ICD) further categorizes febrile seizures into subtypes to enhance diagnostic precision:

1. **Simple febrile seizures (8A63.00):**
   - Generalized tonic-clonic seizures lasting less than 15 minutes.
   - Occur only once within a 24-hour period.
2. **Complex febrile seizures (8A63.01):**
   - Prolonged seizure duration exceeding 15 minutes.
   - May exhibit focal neurological symptoms.
   - Can recur multiple times within 24 hours.
3. **Other specified febrile seizures (8A63.0Y):**
   - Includes febrile seizures not fitting the criteria for simple or complex types.
4. **Unspecified febrile seizures (8A63.0Z):**
   - Reserved for cases where detailed diagnostic information is unavailable.

**Clinical Context and Exclusions**

Febrile seizures are explicitly distinguished from other seizure-related conditions. According to the ICD, the following are excluded from the classification of febrile seizures:

- **Migraine aura-triggered seizures (8A80.3)**
- **Syncope (MG45)**
- **Endocrine, nutritional, or metabolic diseases (05)**
- **Complications of pregnancy, childbirth, and the puerperium (18)**
- **Conditions originating in the perinatal period (19)**
- **Injuries, poisonings, or external causes (22)**

Additionally, seizures occurring in neonates are categorized separately under **neonatal seizures (KB06)**.

Accurate classification of febrile seizures is critical for understanding their epidemiology, clinical management, and genetic predispositions. Subtypes such as **generalized epilepsy with febrile seizures plus (GEFS+)** and **familial mesial temporal lobe epilepsy with febrile seizures** highlight the importance of genetic and syndromic associations. Further, the ICD framework facilitates targeted research, allowing better characterization of these subtypes and their impact on childhood health outcomes. Proper documentation and reporting mechanisms, such as those within the FeverApp-Registry, are essential for advancing research and improving patient care in this domain.^[[2]](#footnote-2)^

1. URL: <https://icd.who.int/browse/2024-01/mms/en#1766832202> [↑](#footnote-ref-1)
2. Nguyen, L., Whitehall, J., & Edwards, M. (2017). Accuracy of clinical coding for febrile seizures and implications for activity‐based funding. *Internal Medicine Journal*, *47*(S3), 21–21. https://doi.org/10.1111/imj.11_13462 [↑](#footnote-ref-2)
